# Impaired human immunodeficiency virus type 1 replicative fitness in atypical viremic non-progressor individuals

**DOI:** 10.1186/s12981-017-0144-0

**Published:** 2017-03-20

**Authors:** Jan Weber, Richard M. Gibson, Lenka Sácká, Dmytro Strunin, Jan Hodek, Jitka Weberová, Marcela Pávová, David J. Alouani, Robert Asaad, Benigno Rodriguez, Michael M. Lederman, Miguel E. Quiñones-Mateu

**Affiliations:** 1 Institute of Organic Chemistry and Biochemistry v.v.i., Academy of Sciences of the Czech Republic, Flemingovo n. 2, 166 10 Prague 6, Czech Republic; 2 University Hospital Translational Laboratory, University Hospitals Cleveland Medical Center, Cleveland, OH USA; 3 Department of Medicine, Case Western Reserve University/University Hospitals Cleveland Medical Center, 10900 Euclid Avenue, Cleveland, OH 44106-7288 USA; 4 Department of Pathology, Case Western Reserve University, Cleveland, OH USA

**Keywords:** HIV-1, Replicative fitness, Disease progression, Viremic non-progressors

## Abstract

**Background:**

Progression rates from initial HIV-1 infection to advanced AIDS vary significantly among infected individuals. A distinct subgroup of HIV-1-infected individuals—termed viremic non-progressors (VNP) or controllers—do not seem to progress to AIDS, maintaining high CD4^+^ T cell counts despite high levels of viremia for many years. Several studies have evaluated multiple host factors, including immune activation, trying to elucidate the atypical HIV-1 disease progression in these patients; however, limited work has been done to characterize viral factors in viremic controllers.

**Methods:**

We analyzed HIV-1 isolates from three VNP individuals and compared the replicative fitness, near full-length HIV-1 genomes and intra-patient HIV-1 genetic diversity with viruses from three typical (TP) and one rapid (RP) progressor individuals.

**Results:**

Viremic non-progressors and typical patients were infected for >10 years (range 10–17 years), with a mean CD4^+^ T-cell count of 472 cells/mm^3^ (442–529) and 400 cells/mm^3^ (126–789), respectively. VNP individuals had a less marked decline in CD4^+^ cells (mean −0.56, range −0.4 to −0.7 CD4^+^/month) than TP patients (mean −10.3, −8.2 to −13.1 CD4^+^/month). Interestingly, VNP individuals carried viruses with impaired replicative fitness, compared to HIV-1 isolates from the TP and RP patients (p < 0.05, 95% CI). Although analyses of the near full-length HIV-1 genomes showed no clear patterns of single-nucleotide polymorphisms (SNP) that could explain the decrease in replicative fitness, both the number of SNPs and HIV-1 population diversity correlated inversely with the replication capacity of the viruses (*r* = −0.956 and *r* = −0.878, *p* < 0.01, respectively).

**Conclusion:**

It is likely that complex multifactorial parameters govern HIV-1 disease progression in each individual, starting with the infecting virus (phenotype, load, and quasispecies diversity) and the intrinsic ability of the host to respond to the infection. Here we analyzed a subset of viremic controller patients and demonstrated that similar to the phenomenon observed in patients with a discordant response to antiretroviral therapy (i.e., high CD4^+^ cell counts with detectable plasma HIV-1 RNA load), reduced viral replicative fitness seems to be linked to slow disease progression in these antiretroviral-naïve individuals.

**Electronic supplementary material:**

The online version of this article (doi:10.1186/s12981-017-0144-0) contains supplementary material, which is available to authorized users.

## Background

Infection with human immunodeficiency virus type 1 (HIV-1)—in the absence of antiretroviral therapy—usually follows a well-defined path of virological and immunological changes; however, progression rates to advanced AIDS vary significantly among infected individuals [1, 2]. Based on plasma HIV-1 RNA (viral) load, CD4^+^ T-cell counts, and symptomatic or asymptomatic HIV-1 infection adult patients have been classified into four groups: (i) rapid progressors (RP), (ii) typical progressors (TP), and two groups of long-term non-progressors (LTNP) namely (iii) elite controllers and (iv) viremic controllers [3, 4]. Among the latter, a rare subgroup of HIV-1-infected individuals has been described who do not seem to progress to AIDS, maintaining high CD4^+^ T-cell counts despite high levels of viremia (i.e., over 2000 HIV-1 copies RNA/ml of plasma) for many years, also called viremic non-progressors (VNPs) [5–8].

Multiple studies have tried to associate these differences in HIV-1 disease progression with a multitude of host (HIV-1-specific immune response and genetic markers) and viral factors [9–13]. Among the immunological factors, strong HIV-1-specific cytotoxic T cell (CTL) responses have been shown to control HIV-1 infection right after transmission, perhaps determining viral set point in chronic stages of infection, which may help reduce the rate of HIV-1 disease progression [14, 15]. Similarly, a series of host genetic factors that seem to influence HIV-1 progression rates have been well described, including human leukocyte antigen (HLA) class I alleles such as HLA-B*27 and HLA-B*57 [16, 17] and a 32 bp deletion in the CCR5 chemokine receptor gene [18–22]. On the other hand, the impact of restriction cellular factors like APOBEC3, TRIM5α, tetherin, and SAMHD1 on HIV-1 progression is still debated [10, 23]. Altogether, it has been long recognized that immune activation is one of the major contributors to HIV-1 disease and pathogenesis [6, 24–27].

Viral factors—such as impaired HIV-1 replicative fitness—have been associated with slow or limited HIV-1 disease progression [11, 28–31], particularly in patients infected with heavily mutated drug resistant viruses [28, 30, 32–35]. High fitness of transmitted HIV-1 has been related to rapid disease progression [36], while some individuals carrying viruses with decreased replication capacity during acute/early infection have been classified as HIV controllers [37]. Although isolating replication competent HIV-1 strains from elite controllers has been difficult, two studies failed to identify any major defect on the replication ability of these viruses [38, 39]. In contrast, recombinant viruses constructed with HIV-1 *gag*, *pol*, and/or *env* genes from elite controllers exhibited reduced replication capacity compared to recombinant viruses constructed from typical progressors [40–42]. On the other hand, very limited information about the replicative fitness of viruses infecting viremic non-progressor individuals is currently available [6, 43]. If a decrease in HIV-1 replicative fitness is associated with slower/no disease progression, what is causing the virus to have this reduced replication capacity—in the absence of drug resistance mutations—in a certain number of non-progressor patients?

To date, no common pattern or polymorphism(s) shared among HIV-1 variants infecting non-progressor individuals have been clearly defined. Several studies have described changes in the LTR, e.g., in the Sp1 binding site [44] or large deletions [11, 45] that could be associated with slow disease progression. Detrimental mutations in Gag have been associated with viral attenuation [46, 47], which seem to play an important role in HIV-1 disease [48, 49]. Significant replicative fitness loss was observed in recombinant viruses encoding *pol* genes with mutations affecting HLA-A, but surprisingly not HLA-B, binding [50], although the most attenuated viruses were those constructed from HIV-1 elite controllers expressing HLA-B*57 and HLA-B*51 [40]. Changes in the V2 region of gp120 [51, 52] and a single amino acid deletion in gp41 [53] in the *env* gene have also been associated with slow disease progression. However, most of the changes in the HIV-1 genome linked to altered pathogenesis have been identified in accessory genes. Single amino acid substitutions [54, 55] and large deletions [56, 57] in Nef have been associated with a decrease in replicative fitness and slow disease progression. Mutations at positions 72 and 77 in Vpr, disrupting the viral protein function, were frequently detected in non-progressors [58, 59]. Finally, single or large insertions in Vpu [60] and amino acid substitutions in Vif [61, 62] have also been associated with non-progression in HIV-1 disease.

In this pilot study we quantified the replicative fitness of HIV-1 isolates obtained from extremely rare viremic non-progressor HIV-infected individuals and compared them to the fitness of those from patients with typical or rapid disease progression. Using deep sequencing we analyzed the full-length HIV-1 genomes and determined intrapatient HIV-1 diversity to investigate if these viral factors could be contributing to the maintenance of stable CD4^+^ T-cell counts despite persistence viremia for more than 10 years, in the absence of antiretroviral treatment.

## Methods

### Clinical samples

Blood samples were obtained during routine patient monitoring from a well-characterized cohort of HIV-infected individuals at the Special Immunology Unite (SIU) at Case Western Reserve University/University Hospitals Cleveland Medical Center (CWRU/UHCMC), with the written informed consent. Seven subjects were selected and grouped based on the following definitions: (i) *viremic non*-*progressors* (VNP, n = 3) corresponding to patients serologically proven to be HIV-1-infected for at least 10 years, CD4^+^ T-cell decline of <45 cells/mm^3^ per year and repeated plasma HIV-1 RNA load >1000 copies/ml in the absence of antiretroviral therapy; (ii) *typical progressors* (TP, n = 3) corresponding to patients serologically proven to be HIV-1-infected for at least 10 years, CD4^+^ T-cell decline of >75 cells/mm^3^ per year and repeated plasma HIV-1 RNA load >1000 copies/ml in the absence of antiretroviral therapy; and (iii) *rapid progressor* (RP, n = 1) corresponding to a patient serologically proven to be HIV-1-infected for at least 5 years, CD4^+^ T-cell decline of >77 cells/mm^3^ per year and repeated plasma HIV-1 RNA load >10,000 copies/ml in the absence of antiretroviral therapy. In the case of the VNP and TP patients, CD4^+^ T-cell numbers and plasma HIV-1 load levels were monitored for a minimum of 30 months (18 months for the RP patient) with at least 10 determinations over this period. Demographics, clinical and virological characteristics are summarized in Table 1.

**Table 1:** Demographic, clinical and virological parameters

Patient ID	Viremic non-progressors (VNP)			Typical progressors (TP)			Rapid progressor (RP)
	VNP-1	VNP-2	VNP-3	TP-1	TP-2	TP-3	RP
Demographics							
Age^a^	44	50	42	40	43	38	30
Sex^b^	M	M	M	M	M	M	M
Race	Black	Caucasian	Black	Caucasian	Black	Black	Black
Risk Factor^c^	MSM	Unknown	MSM	MSM	MSM	MSM	MSM
Years HIV+^d^	14	17	10	14	15	12	5
Follow up (months)^e^	84	37	123	35	19	31	18
CD4^+^ (cells/mm^3^)							
CD4^+^ count^f^	563	443	479	386	361	126	11
Mean CD4^+^ count^g^	529	445	442	483	457	293	57
Range CD4^+^ count^g^	372–711	316–608	280–814	220–789	361–645	126–511	11–124
Slope CD4^+^ count^h^	−0.6	−0.7	−0.4	−9.6	−8.2	−13.1	−4.7
HIV-1 RNA (log 10 copies/ml)							
HIV-1 RNA^f^	5.46	4.90	4.70	4.41	4.27	4.49	4.64
Mean HIV-1 RNA^g^	4.68	4.91	4.31	4.23	4.45	4.67	4.49
Range HIV-1 RNA^g^	3.61–5.45	4.27–5.08	3.62–4.71	3.89–4.62	4.28–4.62	3.90–4.90	4.09–4.66
Virus characteristics							
HIV-1 subtype^i^	B	B	B	B	B	B	B
HIV-1 coreceptor tropism^j^	R5	R5	R5	R5	R5	R5	D/M

*R5* CCR5-tropic virus, *D/M* dual- or mixed-tropic virus

^a^ Age at the time of sampling

^b^ *M* male

^c^ *MSM* men who have sex with men

^d^ Years since first HIV-seropositive test to time of blood sample collection for this study

^e^ Number of months that the patient had been monitored up to the blood sample collection date

^f^ CD4^+^ T-cell count (cells/mm^3^) and HIV-1 RNA plasma load (log10 copies/ml) at the time the blood sample was obtained

^g^ Mean and range CD4^+^ T-cell count and HIV-1 RNA plasma load values determined during the clinical follow up time, to the time of blood sample collection for this study and prior to the initiation of antiretroviral treatment

^h^ Rate of CD4^+^ T-cell count decline (slope) calculated as cells/mm^3^ per month, using all CD4^+^ cell measurements available at the time the blood sample was obtained

^i^ HIV-1 subtype determined using the near full-length HIV-1 genome consensus sequences with the Recombinant Identification Program (RIP) from the Los Alamos HIV Sequence Database (https://www.hiv.lanl.gov/content/sequence/RIP/RIP.html) [76]

^j^ HIV-1 coreceptor tropism determined using sequencing reads corresponding to the V3 region of gp120, *env* gene with the DEEPGEN™HIV Software Tool Suite [70]

### Cells and viruses

Peripheral blood mononuclear cells (PBMC), obtained from HIV-seronegative donors, were stimulated with 2.5 µg/ml of phytohemagglutinin (PHA; Gibco BRL) and maintained in RPMI 1640/2 mM l-glutamine media (Cellgro; Mediatech, Herndon, VA) supplemented with 10% fetal bovine serum (Cellgro), 10 mM HEPES buffer (N-2-hydroxyethylpiperazine-N-2-ethanesulfonic acid; Cellgro), 1 ng/ml of interleukin-2 (IL-2) (Gibco, BRL), 100 U of penicillin/m (Cellgro) and 100 μg of streptomycin/ml (Cellgro), for three days before infection with HIV-1 [28]. HIV-1 isolates were obtained from all seven patients by co-cultivating their PBMCs with PBMCs from HIV-seronegative donors as described [28]. Three primary HIV-1 isolates (HIV-1_A-92UG029_, HIV-1_B-92US076_, and HIV-1_AE-CMU06_) were obtained from the AIDS Research and Reference Reagent Program and propagated in PHA-stimulated, IL-2-treated PBMCs. Tissue culture dose for 50% infectivity (TCID_50_) was determined for each virus in PBMCs in triplicate with serially diluted stocks, based on the reverse transcriptase (RT) activity in culture supernatants on day 7 of culture, using the Reed and Muench method [63]. Viral titers were expressed as infectious units per milliliter (IU/ml).

### HIV-1 replicative fitness determination using viral growth kinetics analysis

The ability of the seven patient-derived HIV-1 isolates, plus the primary wild-type HIV-1_B-92US076_ isolate used as control, to replicate in the absence of drug pressure was determined by measuring viral growth kinetics as described [64, 65]. Briefly, 1 × 10^6^ PHA-stimulated, IL-2-treated PBMCs were infected at a multiplicity of infection (MOI) of 0.001 IU/cell in 1 ml of RPMI 1640 medium and incubated for 2 h at 37 °C in 5% CO_2_. HIV-infected cells were then washed twice with 1x PBS and split to be cultured in triplicate wells of a 96-well plate (1 × 10^5^ cells/well). Fresh PHA-stimulated, IL-2–treated PBMCs (5 × 10^4^ cells) were added to each well at days 5 and 10 post-infection. Reverse transcriptase activity in the culture supernatant was assayed on days 0, 4, 6, 8, 11, and 14 post-infection as described [28]. Viral replication was quantified using the slope of the growth curves and performing linear regression analysis derived from the equation *log(y)* = *mt* + *log(h*), where *y* is virus quantity (cpm), *t* is time in days, and *h* is the *y*-intercept (day 0). All slope values for each virus were used to calculate means and standard deviations. Differences in the mean values were evaluated using a one way analysis of variance test and the significance difference from the control HIV-1_B-92US076_ calculated using the Bonferroni’s Multiple Comparison Test (GraphPad Prism v.6.0b, GraphPad Software).

### HIV-1 replicative fitness determination using growth competition experiments

Dual infection/competition experiments were carried out as previously described [28, 35, 66, 67]. Briefly, each query virus (patient-derived subtype B HIV-1 isolates and the HIV-1_B-92US076_ control) was competed against two different non-subtype B HIV-1 control strains (HIV-1_A-92UG029_ and HIV-1_AE-CMU06_) in a 1:1 initial proportion using an MOI of 0.001 IU/cell to infected 1 × 10^6^ PBMCs for 2 h at 37 °C and 5% CO_2_. Cells were subsequently washed twice with 1× PBS and cultured in a 24-well plate. Cell-free supernatant and cells were harvested at day 10 and stored at −80 °C for subsequent analysis. The final proportions of the two viruses in each competition were quantified using a TaqMan Real-Time PCR assay after normalizing to viral production in the HIV-1 monoinfections as described [28, 35, 67]. Replicative fitness for each patient-derived HIV-1 isolate was calculated and expressed as a percentage of the replicative fitness of the HIV-1_B-92US076_ control, set as 100%.

### Reverse transcription (RT)-PCR amplification of nearly full-length HIV-1 genome

Plasma viral RNA was purified from pelleted virus particles by centrifuging one milliliter of plasma at 20,000*g* × 60 min at 4 °C, removing 860 µl of cell-free supernatant and resuspending the pellet in the remaining 140 µl, to finally extract viral RNA using QIAamp Viral RNA Mini kit (Qiagen; Valencia, CA). Viral RNA was reverse-transcribed using AccuScript High Fidelity Reverse Transcriptase (Stratagene Agilent; Santa Clara, CA) and five previously described antisense external primers (Pan-HIV-1-1R [68], 1R, 2R, 3R, and 4R [69]) in 20 µl reaction mixtures containing 1 mM dNTPs, 10 mM DTT and 10 units of RNAse inhibitor. Six overlapping fragments, covering almost the entire HIV-1 genome, were amplified using a series of external and nested primers and Phusion High-Fidelity DNA Polymerase (NEB, Ipswich, MA) with defined cycling conditions as previously described [68, 69], i.e., 5′LTR (675 bp; HXB2 coordinates 120 to 794), 5′LTR-*gag*/p7 (1269 bp; 658 to 1924), *gag*/p24-*pol*/RT (2327 bp; 1137 to 3463), *pol*/RT-*vif* (2259 bp; 2976 to 5234), *pol*/int-*env*/gp120 (2921 bp; 4602 to 7522), and *env*/gp120-3′LTR (2587 bp; 6858 to 9444).

### Population (Sanger) sequence analysis

Nested PCR products were purified with the QIAquick PCR Purification kit (Qiagen) and sequenced by Sanger (population) sequence (GATC Biotech, Constance, Germany). Nucleotide sequences were analyzed using DNASTAR Lasergene Software Suite v.12.3.1.4 (Madison, WI).

### Deep sequencing of nearly full-length HIV-1 genome

All six overlapping HIV-1 fragments, from the seven patient-derived plasma samples, were deep sequenced using a variation of the DEEPGEN™HIV assay [70]. Briefly, the six amplicons were purified (Agencourt AMPure XP, Beckman Coulter) and quantified (2100 Bioanalyzer DNA 7500, Agilent Technologies) prior to using the Ion Xpress Fragment Library Kit (Life Technologies, Carlsbad CA) to construct a multiplexed library for shotgun sequencing on the Ion Personal Genome Machine (PGM, Life Technologies). For that, a mixture of all six purified DNA amplicons (16 ng each) was randomly fragmented and blunt-ends repaired using the Ion Shear Plus Reagent (Life Technologies) followed by DNA purification (Agencourt AMPure XP, Beckman Coulter). The P1 adapter and one of seven barcodes were ligated to the repaired fragment ends prior to DNA purification (Agencourt AMPure XP, Beckman Coulter). DNA fragments were then selected by size (i.e., 280–320 bp; Pippin Prep, Life Technologies) and each barcoded library, i.e., a mixture of all six amplicons per sample, was purified (Agencourt AMPure XP, Beckman Coulter) and normalized using the Ion Library Equalizer Kit (Life Technologies). All seven barcoded DNA libraries, corresponding to seven patient-derived amplicons, were pooled in equimolar concentrations and templates prepared and enriched for sequencing on the Ion Sphere Particles (ISPs) using the Ion OneTouch 200 Template Kit v2 (Life Technologies) in the Ion OneTouch 2 System (Life Technologies). Templated ISPs were quantified (Qubit 2.0, Life Technologies) and loaded into an Ion 318 Chip (Life Technologies) to be sequenced on the Ion PGM using the Ion PGM Sequencing 200 Kit v2 (Life Technologies). Sequencing run, signal processing and base calling was performed with Torrent Analysis Suite version 5.0.4. All deep sequencing experiments were performed in the CLIA/CAP-accredited University Hospitals Translational Laboratory under a good laboratory practice framework.

### Read mapping, variant calling, phylogenetic and viral diversity analysis

Reads were mapped and aligned (assembled) against the HIV-1_HXB2_ (GenBank: K03455) reference sequence using SeqMan NGen (DNASTAR Lasergene Software Suite v.12.3.1.4), and the assemblies analyzed using SeqMan Pro (DNASTAR Lasergene Software Suite v.12.3.1.4). Full-length HIV-1 consensus sequences were generated for each patient-derived virus, compared to the corresponding population sequences obtained by Sanger sequencing, aligned using ClustalW [71] and their phylogeny reconstructed using the neighbor-joining statistical method as implemented within MEGA 6.06 [72]. Variant calling (i.e., single nucleotide polymorphisms, including substitutions, deletions and insertions) and their frequencies in the virus population relative to the HIV-1_HXB2_ reference sequence were quantified using a proprietary pipeline (Alouani and Quiñones-Mateu, unpublished results). Intra-patient HIV-1 quasispecies diversity was determined using near full-length HIV-1 genome (or individual genes and coding region sequences) based on the p-distance model as described for deep sequencing [73].

### Statistical analyses

Descriptive results are expressed as mean values, standard deviations, range, and confidence intervals. As described above, differences in the mean of the slope values for the viral growth kinetics curves were determined using a One Way Analysis of Variance test and the difference from the reference HIV-1_NL4-3_ virus calculated using the Bonferroni’s Multiple Comparison Test. All differences with a *P* value of <0.05 were considered statistically significant. All statistical analyses were performed using GraphPad Prism v.6.0b (GraphPad Software, La Jolla, CA) unless otherwise specified. Nucleotide sequences of the nearly full-length HIV-1 genomes, both consensus and individual reads, obtained by deep sequencing in this study have been submitted to the Los Alamos National Laboratory HIV-DB Next Generation Sequence Archive (http://www.hiv.lanl.gov/content/sequence/HIV/NextGenArchive/Weber2017).

## Results

### Clinical, virologic, and immunologic characteristics of viremic non-progressors, typical and rapid progressor patients

As described above, for this study we identified three treatment-naïve HIV-infected individuals classified as VNPs. At the time of the study, these patients were infected with HIV-1 for over 10 years (range 10–17 years), maintaining relatively high CD4^+^ T-cell counts (mean 472, range 280–814 cells/mm^3^) with limited decline in CD4^+^ T-cell count (mean slope −0.56, range −0.4 to −0.7 CD4^+^ T-cells/month) despite sustained plasma HIV-1 RNA loads above 4000 copies/ml (mean 5.02 log_10_, range 3.61–5.45 copies/ml) during the time these patients were monitored prior to this study (range 37–123 months) (Table 1; Additional file 1: Figure S1). For comparison, we selected three patients classified as typical progressors (each infected for at least 10 years, range 12–15 years), and one rapid progressor infected for 5 years. Overall, the CD4^+^ T-cell counts for the TPs were similar to that of the VNPs (mean 400, range 126–789 cells/mm^3^) albeit with a more marked decline in CD4^+^ T-cells over time (mean slope −10.3, range −8.2 to −13.1 CD4^+^ T-cells/month). Plasma HIV-1 RNA levels for the TPs were similarly high (mean 4.45 log_10_, range 3.89–4.9 copies/ml). As expected, the CD4^+^ T-cell counts from the RP patient were significantly lower than those for the VNP and TP individuals (mean 57, range 11–124 cells/mm^3^), declining at a rate of −4.7 CD4^+^ T-cells/month with high plasma HIV-1 RNA loads (mean 4.49 log_10_, range 4.09–4.66 copies/ml) (Table 1; Additional file 1: Figure S1). The rest of the clinical baseline characteristics showed no significant differences regarding age, gender distribution, ethnicity, and mode of transmission of HIV-1.

### Replicative fitness of HIV-1 isolates from VNP, TP, and RP patients

**Fig. 1:**
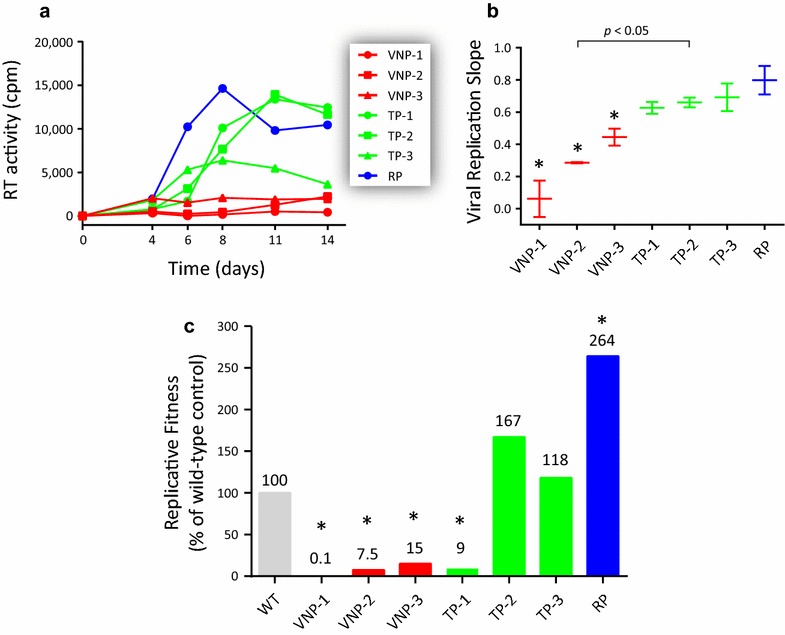
Replicative fitness of seven patient-derived HIV-1 isolates in the absence of any host selective pressure, including antiretroviral drugs. **a** Seven HIV-1 isolates from 3 viremic non-progressor (VNP), 3 typical (TP) and one rapid (RP) progressor patients were evaluated for their ability to replicate in peripheral blood mononuclear cells (PBMC). Virus replication was quantified by measuring reverse transcriptase (RT) activity in the cell-free supernatant. **b** Viral replication slopes were calculated using the slopes between cpm values at days 0 and 4, 0 and 6, 0 and 8, 0 and 11, and 0 and 14. All five slope values for each virus were used to calculate the mean, standard deviation, and 10th and 90th percentiles. Differences in the mean values were calculated using a One Way Analysis of Variance test and the significance difference from the primary wild-type HIV-1_B-92US076_ control isolate calculated using the Bonferroni’s Multiple Comparison Test. **c** Each patient-derived HIV-1 isolate was competed against two different non-subtype B HIV-1 control strains (HIV-1_A-92UG029_ and HIV-1_AE-CMU06_) in PBMCs and their replicative fitness calculated and expressed as a percentage of the replicative fitness of the reference virus HIV-1_B-92US076_ (set as 100%) as described [28, 35, 66, 67]. Values represent results obtained from single growth competition experiments. Viral replication kinetics and growth competitions marked with an *asterisk* were significantly different to the HIV-1_B-92US076_ control (p < 0.05, 95% CI)

We used two different but complementary approaches to quantify the ability of all seven patient-derived HIV-1 isolates (i.e., 3 VNPs, 3 TPs, and 1 RP) to replicate in vitro in the absence of any host selective pressure, including antiretroviral drugs. A first glimpse of the replicative fitness of these viruses was obtained using classical viral growth kinetics in PBMCs from HIV-seronegative donors (Fig. 1a). Statistical analysis of the slope of the growth curves showed that the replicative fitness of all three VNP viruses was significantly reduced compared to the replicative fitness of the three TP and RP viruses (p < 0.05, 95% CI, Fig. 1b). Since in vitro dual infection/growth competition experiments are considered the gold standard method to measure viral fitness [30, 74] we competed each one of the seven HIV-1 isolates against two different non-subtype B HIV-1 control strains (HIV-1_A-92UG029_ and HIV-1_AE-CMU06_). Interestingly, all three VNP viruses showed a marked decrease in replicative fitness, i.e., 0.1, 7.5, and 15% relative to that of the primary HIV-1_B-92US076_ isolate used as control (Fig. 1c). With the exception of the TP-1 virus (9%), the replicative fitness of the other two TP viruses was not impaired relative to the fitness of the HIV-1_B-92US076_ control (167 and 118% for TP-2 and TP-3, respectively). Finally, the RP virus had a 2.6-fold higher replicative fitness than the HIV-1_B-92US076_ control and, in average, was 35-fold more fit to replicate in PBMCs than the VNP viruses (Fig. 1c).

### Characterization of HIV-1 isolates from VNP, TP, and RP individuals: subtyping, drug resistance, and coreceptor tropism

**Fig. 2:**
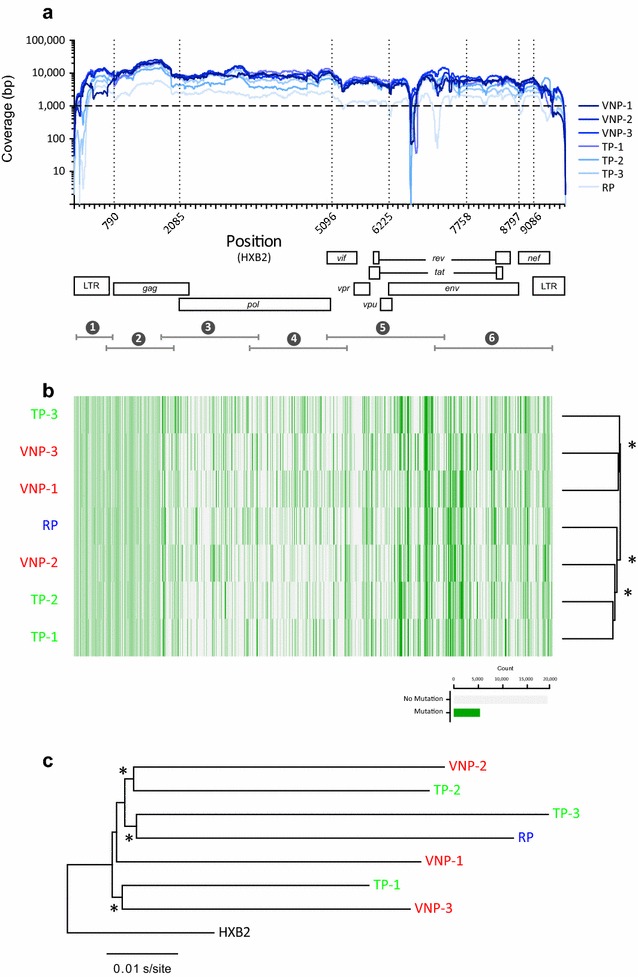
**a** Coverage, i.e., number of reads per nucleotide position, obtained by deep sequencing the seven patient-derived HIV-1 isolates. Near full-length HIV-1 genomes were RT-PCR amplified and deep sequenced as described in “Methods”. The position relative to the HIV-1 genome of the six overlapping amplicons used to amplify and sequence the near full-length HIV-1 genomes (HXB2 position 157 to 9428, not counting primer sequences) is indicated. **b** Hierarchical clustering analysis of the single nucleotides polymorphisms (SNPs) was used to group the seven patient-derived HIV-1 isolates by similarity. Dendrograms were calculated using the Euclidean distance and Complete cluster methods with 1000 bootstrap iterations as described (http://www.hiv.lanl.gov/content/sequence/HEATMAP/heatmap.html) Bootstrap values >60% are indicated by an *asterisk*. *Green and grey blocks* indicate the presence or absence of SNPs, respectively, in each HIV-1 isolate relative to the HIV-1_HXB2_ reference. **c** A Neighbor-joining phylogenetic tree was constructed using the near full-length HIV-1 consensus sequences generated for each patient-derived virus (obtained from deep sequencing reads) and rooted using the HIV-1_HXB2_ sequence (GenBank accession number AF033819). Bootstrap resampling (1000 data sets) of the multiple alignment tested the statistical robustness of the tree, with percentage values above 75% indicated by an *asterisk*. *s/site* substitutions per nucleotide site

To further characterize the HIV-1 isolates, viral RNA was used to amplify and sequence near full-length HIV-1 genomes (HXB2 coordinates 157–99,428) using both Sanger and deep sequencing. For deep sequencing, amplicons corresponding to all 7 viruses were multiplexed into a single 318c chip (78% loading efficiency of Ion Sphere Particles), generating 3,856,115 total quality reads with a mean read length of 131 bp (mean range 130–133 bp). Although the average sequencing coverage at each nucleotide position varied with each sample and HIV-1 genomic region analyzed (*p* < 0.0001, one-way ANOVA), the mean coverage for each viral sequence ranged from 2079 reads for the RP virus to 8500 for VNP-3 (Fig. 2a). These metrics ensured the minimum coverage of 1000 per nucleotide position sequenced required to guarantee the detection of a minor variant present at least at 1% of the population [75]. As expected, coverage decreases abruptly at the ends of the amplicons (e.g., 5′LTR and 3′LTR in Fig. 2a); however, we observed an inexplicable decrease in coverage around positions 6655–6719 (corresponding to variables regions V1 and V2 in gp120, *env* gene) that do not correspond with the end of any of the overlapping PCR products covering this genomic region.

The entire HIV-1 genome from all seven viruses was classified as subtype B using the Recombinant Identification Program from the Los Alamos HIV Sequence Database [76] (Table 1; Additional file 2: Figure S2). We used our proprietary DEEPGEN™HIV Software Tool Suite [70] to confirm the absence of mutations (at >1% frequency in the HIV-1 population) associated with drug resistance in the protease (PR), reverse transcriptase (RT), and/or integrase (INT) coding regions. The same tool was used to determine HIV-1 coreceptor tropism based on sequencing reads corresponding to the V3 region of gp120. HIV-1 isolates from VNP and TP patients were all CCR5-tropic viruses, while the RP individual was infected with a dual- or mixed-tropic virus (Table 1).

### HIV-1 genetic polymorphisms in VNP, TP, and RP viruses

HIV-1 isolates from viremic non-progressor individuals showed a clear impairment on their ability to replicate in PBMCs in vitro. Since the fitness decrease was not associated with drug resistance mutations, we decided to analyze the entire HIV-1 genomes to try to identify any particular signature(s) that could be responsible for the decrease in replication capacity. First, we scanned all seven near full-length HIV-1 genomes searching for patterns in single-nucleotide polymorphisms (SNPs) among the three groups of patients. As described in Fig. 2b, we observed a higher concentration of SNPs in the variable regions of gp120; however, no clear clustering of the VNP or TP groups was evident when the SNP patterns of the near full-length genomes were analyzed. For example, VNP-1 and VNP-3 clustered with TP-3, while VNP-2 was closer to TP-1 and TP-2. Similar results were observed when SNP patterns were analyzed in each individual HIV-1 genomic region, with the exception of gp41 where significant clustering of VNP-1 with VNP-3 and all three TNP viruses was evident (Additional file 3: Figure S3).

**Fig. 3:**
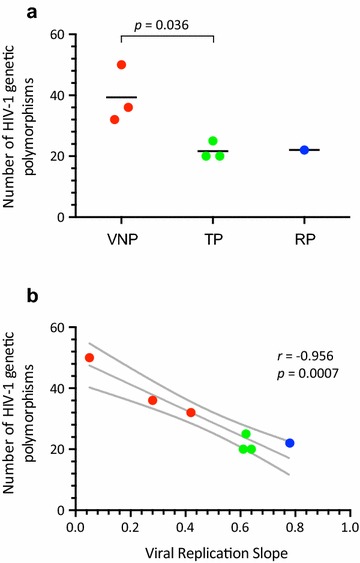
**a** Comparison of the number of HIV-1 genetic polymorphisms in the near full-length HIV-1 genome that have been associated with impaired HIV-1 replicative fitness and/or disease progression [11, 31, 32, 40, 42, 44–62, 77, 78] among the three groups of patients, i.e., VNP, RP, and TP. Unpaired t test was used to assess the statistical significance between VNP and TP patients. A detailed list of the mutations is included in Table 2. **b** Pearson correlation coefficient was used to determine the strength of association between the number of HIV-1 genetic polymorphisms and the HIV-1 replicative fitness calculated by viral growth kinetics analysis (viral replication slope) described in Fig. 1. *r* correlation coefficient, *p* two-tailed p value. *Dotted lines* represent 95% confidence intervals

Next, we focused on HIV-1 genetic polymorphisms that have been associated with impaired HIV-1 replicative fitness and/or disease progression [11, 31, 32, 40, 42, 44–62, 77, 78]. We identified a series of SNPs in LTR and amino acid substitutions—at different frequencies in the virus population—in *gag*, *vif*, *vpr*, *rev*, and *nef* genes (Table 2). Although no clear pattern of signature mutations was shared among the individual viruses from each group, some of the mutations were present only in VNP and not in TP or RP sequences, e.g., in the LTR (insertion at position 329, G364A, C386T, or G399A), *gag* (E12Q, A146P, T242 N, or E482D), *vif* (R132S), *vpr* (F72S), and *nef* (T138C). Interestingly, most of these mutations were identified in the VNP-1 isolate, which had the most impaired replicative fitness of the group (Fig. 1). In fact, the number of HIV-1 genetic polymorphisms previously associated with fitness decrease and/or disease progression was significantly higher in the VNP group compared with the TP and RP viruses (mean 39.3, 21.6, and 22, respectively, *p* = 0.036) (Table 2; Fig. 3a). Moreover, a strong significant inversed correlation was observed between the number of HIV-1 genetic polymorphisms in each viral isolate and the HIV-1 replicative fitness values determined by the slopes of the viral growth curves (*r* = −0.956, *p* = 0.0007, Pearson coefficient correlation) (Fig. 3b). Although not significant, most likely perhaps due to the low fitness calculated for the TP-1 virus, a similar trend was observed using the replicative fitness values from the growth competition experiments (*r* = −0.547, *p* = 0.203, Pearson coefficient correlation).

**Table 2:** HIV-1 genetic polymorphisms identified in this study, which have previously been associated with disease progression and/or impaired replicative fitness

Position^a^	Viremic non-progressors (VNP)			Typical progressors (TP)			Rapid progressor (RP)
	VNP-1	VNP-2	VNP-3	TP-1	TP-2	TP-3	RP
LTR							
287–292	A219G (18%)	A292ins (1%)	C290T (2%)		A219G (3%) A219ins (3%)	T287C (11%) T292A (99%)	
319–324	T319A (99%)	T319A (100%) A324G (100%)	T319A (99%) A324G (27%)	T319A (99%) A324G (99%)	T319A (99%) 320ins (11–57%) A324G (72%)	T319A (99%)	T319A (100%)
325–333	329ins (39%) C333T (11%)	332del (3%)	328ins (1%)		G331A (1%)		
346–356	G350A (1%)		A348T (11%) 355del (2%)	G350A (3%)			
361–371	G363A (6%) G364A (7%)					G366A (1%)	T32C (9%) G363A (10%)
374–385	A374G (74%) A378G (1%) G379A (1%) G385A (97%)	G377A (3%) 381ins (1%)	A374G (7%)	381ins (1%)	A378G (2%) 380ins (1%) C381T (99%) G384A (1%)	374ins (91%) G384A (100%) G385A (100%)	A374G (9%) 374ins (52%) G384A (99%)
386–397	C386T (97%) C387G (2%) G389A (30%)	G395A (2%)	380del (1%)	G389A (2%)		396ins (99%)	387G (3%) G393A (2%)
398–409	G399A (7%)		398ins (2%)	403ins (1%) 405ins (3%)	T398A (90%)	G409A (8%)	G400A (18%)
427–431	430del (1%), 431del (1%)	430del (2%)	430del (9%), 431del (1%)	430del (2%)	431del (2%)		
455–515	11SNPs (>1%), 13 indels (>1%)	8 SNPS, 11 indels	3 SNPs (>1%), 12 indels (>1%)	3 SNPs (>1%), 5 indels (>1%)	2 SNPs (>1%), 6 indels (>1%)	4 SNPs (>1%), 2 indels (>1%)	5 SNPs (>1%), 3 indels (>1%)
*gag*							
823–825	E12Q (99%)						
988–990		S67A (16%)			S67A (3%)	S67A (100%)	
1093–1095	D102E (98%)	D102E (9%)			D102E (73%)		D102E (100%)
1225–1227		A146P (86%)					
1513–1515			T242N (99%)				
1531–1533	G284A (99%)					G284A (99%)	G284A (99%)
1954–1956			T389I (99%)				T389I (100%)
2233–2235		E482D (99%)					
*vif*							
5434–5436	R132S (92%)						
*vpr*							
5773–5775	F72S (4%)						
5788–5790		R77Q (99%)	R77Q (99%)	R77Q (99%)	R77Q (99%)	R77Q (99%)	R77Q (99%)
5806–5826	Ins (7%)						
*rev*							
8522–8524	Q74H (99%)	Q74P (99%)			Q74P (75%)		
8612–8614		V104G (99%)				V104L (33%)	
*nef*							
9208–9210		T138C (99%)					
8839–8841		T15A (99%)	T15A (98%)	T15N (100%)			
8950–8952							
9100–9102	H102Y (99%)	H102Y (99%)	H102W (99%)	H102W (99%)	H102Y (99%)	H102Y (99%)	H102Y (100%)
9340–9342	E182Q (99%)		E182V (99%)	E182V (99%)			

^a^ Positions in the HIV-1 genome relative to the HIV-1_HXB2_ (GenBank: K03455) strain reference. Single-nucleotide polymorphisms (SNPs) in the LTR or amino substitutions in the HIV-1 coding regions are indicated, including their frequency in the virus population (%) quantified by deep sequencing. For example: A219G (18%) in the LTR of the VNP-1 HIV-1 isolate or R77Q (99%) in the *vpr* gene of the VNP-2 HIV-1 isolate. Indels, insertions and/or deletions

### Genetic diversity of VNP, TP, and RP viruses

**Fig. 4:**
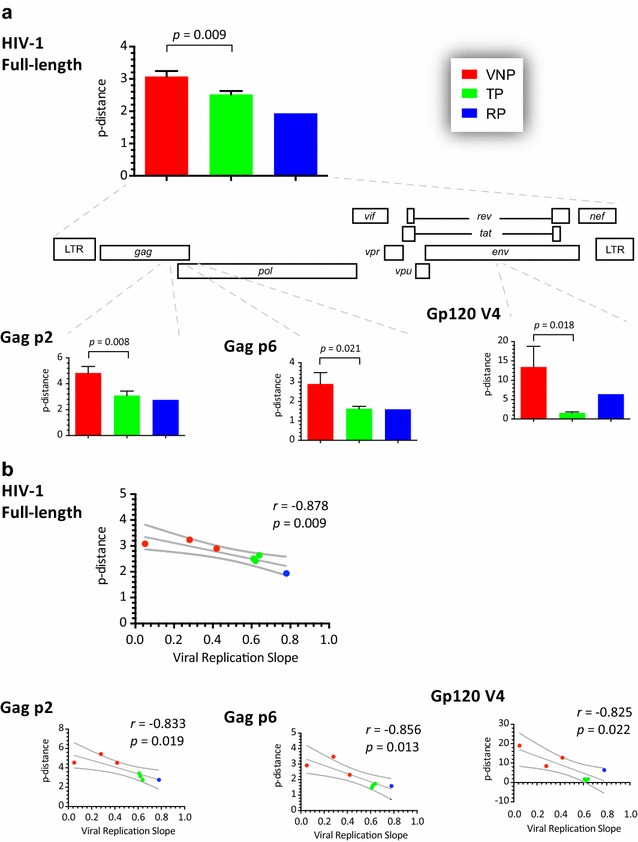
**a** Comparison of intra-patient HIV-1 population diversity, based on the p-distance model [73], among the three groups of patients, i.e., VNP, RP, and TP. Unpaired t test was used to assess the statistical significance between VNP and TP patients. Only those HIV-1 regions significantly different, i.e., near full-length HIV-1 genome, Gag p2, Gag p6, and Gp120 V4, are included. **b** Pearson correlation coefficient was used to determine the strength of association between intra-patient HIV-1 population diversity values (p-distance) and the HIV-1 replicative fitness calculated by viral growth kinetics analysis (viral replication slope) described in Fig. 1. *r* correlation coefficient, *p* two-tailed p value. *Dotted lines* represent 95% confidence intervals

We used the consensus sequences of the near full-length HIV-1 genomes generated by deep sequencing to construct a neighbor-joining phylogenetic tree. As observed in Fig. 2c, the HIV-1 sequences did not cluster together according to their group, i.e., VNP, TP, and RP. Identical results were obtained using the population sequences generated by Sanger sequencing (data not shown). Based on the consensus near full-length HIV-1 sequences, intra-group genetic distances were not significantly different between VNP and TP viruses (0.089 and 0.096 substitutions/site, respectively, *p* = 0.345; Maximum Composite Likelihood model). Next we used the myriad of reads obtained by deep sequencing to calculate intra-patient HIV-1 population diversity based on the p-distance model [73]. Interestingly, the VNP viruses showed significantly higher genetic diversity than the TP and RP viruses (mean 3.07, 2.52, and 1.93 substitutions/site, *p* = 0.009) when the near full-length HIV-1 genomes were analyzed (Fig. 4a). Although this trend was consistent across individual HIV-1 genomic regions and genes (data not shown), the higher genetic diversity in VNP viruses was significant in two regions of the *gag* gene: p2 (4.82, 3.09, and 2.76 substitutions/site, *p* = 0.008) and p6 (2.9, 1.62, and 1.59 substitutions/site, *p* = 0.02), and the V4 region of gp120 (13.4, 1.54, and 6.39 substitutions/site, *p* = 0.018) (Fig. 4a). More importantly, we observed a strongly significant negative correlation between genetic diversity of the near full-length HIV-1 genomes and the replicative fitness values determined by the slopes of the viral growth curves (*r* = −0.878, *p* = 0.009, Pearson coefficient correlation) (Fig. 4b). The same significant inverse associations were observed for the two regions of the *gag* gene: p2 (*r* = −0.833, *p* = 0.019, Pearson coefficient correlation) and p6 (*r* = −0.856, *p* = 0.013, Pearson coefficient correlation), and the V4 region of gp120 (*r* = −0.825, *p* = 0.022, Pearson coefficient correlation) (Fig. 4b) but not for those regions with no significant differences in HIV-1 diversity between the three groups of patients. Similar results, i.e., more heterogeneous virus population (VNPs) having lower viral replicative fitness values, were obtained when replicative fitness was determined using growth competition experiments (regression values ranging from −0.791 to −0.867, *p* < 0.05, Pearson coefficient correlation; data not shown).

## Discussion

Since the discovery of HIV-1 as the etiological agent of AIDS [79, 80], multitude of studies have attempted to understand the differences on HIV-1 disease progression, particularly host and/or viral factors that could be associated with HIV-1 pathogenesis [11, 12, 81]. The common (“normal”) inverse correlation between CD4^+^ T-cell count and plasma HIV RNA load usually governs HIV-1 pathogenesis, i.e., elevated plasma viremia is typically accompanied by a decrease in CD4^+^ T-cells, leading to disease progression [2]. Some HIV-infected individuals have shown uncommon rates of disease progression (slow, non-progressor or elite controllers) but all of them have reduced or undetected plasma HIV RNA loads [3, 4, 42, 82]. On the other hand, a rare group of patients termed viremic non-progressors (VNP) seem to resemble Simian Immunodeficiency Virus (SIV) infection in sooty mangabeys, i.e., active viral replication without (or very limited) disease progression [26, 82–84]. Here we identified three atypically VNP HIV-infected individuals and proceeded to study their HIV-1 isolates, focusing on replicative fitness and viral genomic analysis to try to understand the maintenance of stable CD4^+^ T-cell counts despite persistence viremia for more than 10 years, in the absence of antiretroviral treatment.

As described above, viremic non-progressor (controller) patients do not progress to AIDS, maintaining high CD4^+^ T-cell counts with HIV-1 replication for many years [5–7, 85]. The three VNP individuals described in this study had been infected with HIV-1 for at least 10 years at the time the blood sample was taken, with stable CD4^+^ T-cell counts and high plasma HIV RNA loads. When compared with three typical (TP) and one rapid progressor (RP) patients, the number of CD4^+^ T-cells barely declined in the VNPs over this period of time, similar to what have been described for other adult and children VNPs [43, 82–84, 86]. No other significant differences were observed among the VNP and TP patients, including plasma viral load levels and basic virus characteristics such as HIV-1 subtype (all clade B) and coreceptor tropism (all CCR5-tropic viruses).

Most studies involving the characterization of viremic non-progressor (controller) patients have focused in host factors to try to understand their odd HIV-1 disease progression profile. For example, the presence of the HLA-B*27 allele was associated with the VNP phenotype [43], while a higher frequency of the protective single nucleotide polymorphism −35 CC in the HLA-C gene was found in VNP patients [5]. Preferential Gag-specific CD8^+^ T cell responses have also been observed in viremic controllers [84] and limited infection of CD4^+^ central memory cells [8], perhaps related to low CCR5 expression on these cells [86], seem to be associated with lack of disease progression in VNPs. Limited deregulation of a series of interferon-stimulated genes correlating with time to disease progression was also identified in VNPs [85]. However, and similar to the absence of immune activation during natural SIV infection in sooty mangabeys [27, 87, 88], healthy CD4^+^ T cell counts despite detectable viremia seem to be common in most viremic non-progressor patients [86]. Yet, just a limited number of studies have analyzed HIV-1 strains infecting VNPs [6, 43].

Here we showed that the replicative fitness of all three VNP-derived HIV-1 isolates was impaired compared to the fitness of the viruses obtained from the TP and RP patients. We and others have reported that HIV-1 replicative fitness is associated with disease progression [28–30, 42], that is, viruses with decreased replication capacity influence the natural history of HIV-1 infection, correlating with diminished viral burden and HIV-1 pathogenesis. Viruses from long-term non-progressor [28], slow progressor [41, 42] and elite controller [37, 40, 42] patients have been shown to have impaired replication capacity; however, the fitness of viruses from VNP individuals have not been extensively described. Choudhary et al. [6] showed that HIV-1 isolates derived from VNP were as cytopathic as viruses isolated from typical progressor patients. On the other hand, O’Connell et al. [43] observed a slight decrease in the fitness of a virus from a VNP patient, transmitted from a chronic progressor partner, but failed to identify any amino acid substitution(s) potentially responsible for this phenotype.

Reduced HIV-1 replicative fitness is usually the consequence of changes in the viral quasispecies following the evasion of key selective pressures, e.g., host immune response and/or antiretroviral treatment [30, 31]. These changes are accompanied by single or multiple mutations in the targeted HIV-1 genomic region, deviating the virus population from the wild-type quasispecies distribution and impairing their ability to replicate efficiently compared to the original virus [89, 90]. The effect of a multitude of drug resistance mutations (in the *pol* gene) in viral replicative fitness have been amply described [30, 74]. A number of CTL escape mutations have been identified in HIV and SIV that carry a concomitant replicative fitness cost [46, 91, 92], including HIV-1 mutant variants escaping Gag-specific CD8^+^ T cell responses—like those identified in viremic controllers [84]—that had reduced replicative fitness [40]. Other studies have shown how multiple mutations and/or deletions in the LTR and all HIV-1 coding regions have a range of effects on the ability of the virus to replicate (virus attenuation) [11, 31, 32, 40, 42, 44–62, 78]. Here we used deep sequencing to analyze near full-length HIV-1 genomes from all VNP-derived viruses, and compared them with the sequences from the TP- and RP-derived viral genomes. Despite identifying a series of SNPs in different genomic regions, some of them previously associated with a reduction in replicative fitness and/or disease progression [11, 31, 32, 40, 42, 44–62, 77, 78], no clear pattern of signature mutations was detected in the viruses from the VNPs that could explain the impairment in replication capacity. Interestingly, it was the number of these HIV-1 genetic polymorphisms that correlated significantly with the replicative fitness of each HIV-1 isolate. In fact, it is reasonable to think that viruses with a higher number of critical SNPs have a more marked decrease in replicative fitness, similar to what has been described for multidrug-resistant HIV-1 variants carrying multiple mutations in the protease, reverse transcriptase and/or integrase coding regions [30, 74]. These results were corroborated by calculating intra-patient HIV-1 population diversity, i.e., the highly replication impaired VNP viruses had a more complex virus quasispecies population, most likely as a consequence of trying to evade the host immune response [93], which was highlighted by the significant correlation between replicative fitness and HIV-1 genetic diversity in highly immunogenic regions such as Gag and Gp120 [31, 42, 92]. We and others have described similar results where drug resistant HIV-1 variants with more heterogeneous virus population had lower viral replicative fitness [94, 95]. It is possible that impaired (less replication competent) HIV-1 strains are capable to ascertain a limited host immune response in VNPs, enough to maintain high levels of viremia, resulting in viral evolution but limiting host immune activation that could exacerbate HIV disease, i.e., decrease CD4^+^ T-cell counts. It is also possible that the preferential replication of HIV-1 in effector memory T cells, and preservation of central memory T cells, in VNPs [8] could result in greater virus production per HIV-infected cell. All interesting possibilities to be addressed in further studies.

Although prevalence of elite controllers seems to be less than 1% of the HIV-infected population [96, 97], a recent study involving over 13,000 HIV-infected individuals identified 271 viremic controllers for a 2.03% prevalence [82]. Interestingly, viremic pediatric nonprogressor patients with high CD4^+^ T cell counts seem to be more common than adults VNP individuals [86]. Is it possible that VNP patients are more common than what we originally thought? If so, what can we learn from these individuals that could help monitor and control HIV-1 infection in less fortunate patients? Several studies have pointed to high levels of immune activation as the major cause of HIV-1 pathogenesis [24, 98] and low immune activation seems to be—at least in part- responsible for the lack of disease progression in VNP individuals [86]. Moreover, the VNP-like phenotype during natural SIV infections in sooty mangabeys is definitely not related to a low rate of viral production nor an impairment on replicative fitness of the virus [25, 87]. However, we cannot rule out that original infection with less replication-competent viruses (therefore seeding HIV-1 reservoirs with replication impaired variants) could contribute to the decrease in immune activation, leading to the viremic non-progressor phenotype in these patients. Here we observed that all three VNP patients carried HIV-1 isolates with decreased replicative fitness compared to typical and rapid progressor individuals, and that accumulation of single-nucleotide polymorphisms (mutations) across the HIV-1 genome may be contributing to the overall fitness impairment. Therefore, it is possible that similar to the phenomenon observed in patients with a discordant response to antiretroviral therapy, i.e., high CD4^+^ cell counts with detectable plasma HIV-1 RNA load [99, 100], reduced viral replicative fitness could be contributing to late disease progression in untreated viremic controller individuals. Additional studies involving host immunology and genetics, together with more detailed analysis of the HIV-1 strains infecting VNP patients, are necessary to better understand HIV-1 pathogenesis, including approaches to prevent HIV-1 disease and potentially eliminate HIV reservoirs.

## Additional files

**Additional file 1: Figure S1.** CD4^+^ T-cell counts and plasma HIV-1 RNA load values available for all seven patients described in this study. Vertical dashed lines indicate the time of sampling. Grey arrows depict the number of years since primary HIV-1 infection. The period of time the patients were under antiretroviral treatment (ART) is indicated by black boxes.

**Additional file 2: Figure S2.** Subtyping of the near full-length HIV-1 consensus sequences, obtained by deep sequencing, was performed using the Recombination Identification Program (RIP 3.0) from the Los Alamos HIV Sequence Database as described (1). The similarity plots show distance measurements between the query sequence (patient-derived HIV-1) and the reference HIV-1 sequences from different subtypes.

**Additional file 3: Figure S3.** Hierarchical clustering analysis of single nucleotide polymorphisms (SNPs) in all HIV-1 coding regions plus the long-terminal regions (LTRs). Dendograms were calculated using the Euclidian distance and Complete cluster methods with 1000 bootstrap iterations as described in Los Alamos HIV Sequence Database (http://www.hiv.lanl.gov.content/sequence/HEATMAP/heatmap.html). Bootstrap values >60% are indicated by an asterisk. Green and grey blocks indicate the presence and absence of SNPs, respectively.

## Abbreviations

HIV-1: human immunodeficiency virus type 1
VNP: viremic non-progressors
TP: typical progressor
RP: rapid progressor
LTNP: long-term non-progressors
SNP: single-nucleotide polymorphism
HLA: human leukocyte antigen
CTL: cytotoxic T cell
PBMC: peripheral blood mononuclear cells
MOI: multiplicity of infection
