# Supplementary material for: Impaired human immunodeficiency virus type 1 replicative fitness in atypical viremic non-progressor individuals
Source: AIDS Res Ther. 2017 Mar 20;14:15. doi: 10.1186/s12981-017-0144-0 (PMC5359922; doi:10.1186/s12981-017-0144-0)
Supplement: Supplementary file 1 — Additional file 1: Figure S1. CD4+ T-cell counts and plasma HIV-1 RNA load values available for all seven patients described in this study. Vertical dashed lines indicate the time of sampling. Grey arrows depict the number of years since primary HIV-1 infection. The period of time the patients were under antiretroviral treatment (ART) is indicated by black boxes. [file 12981_2017_144_MOESM1_ESM.pdf]

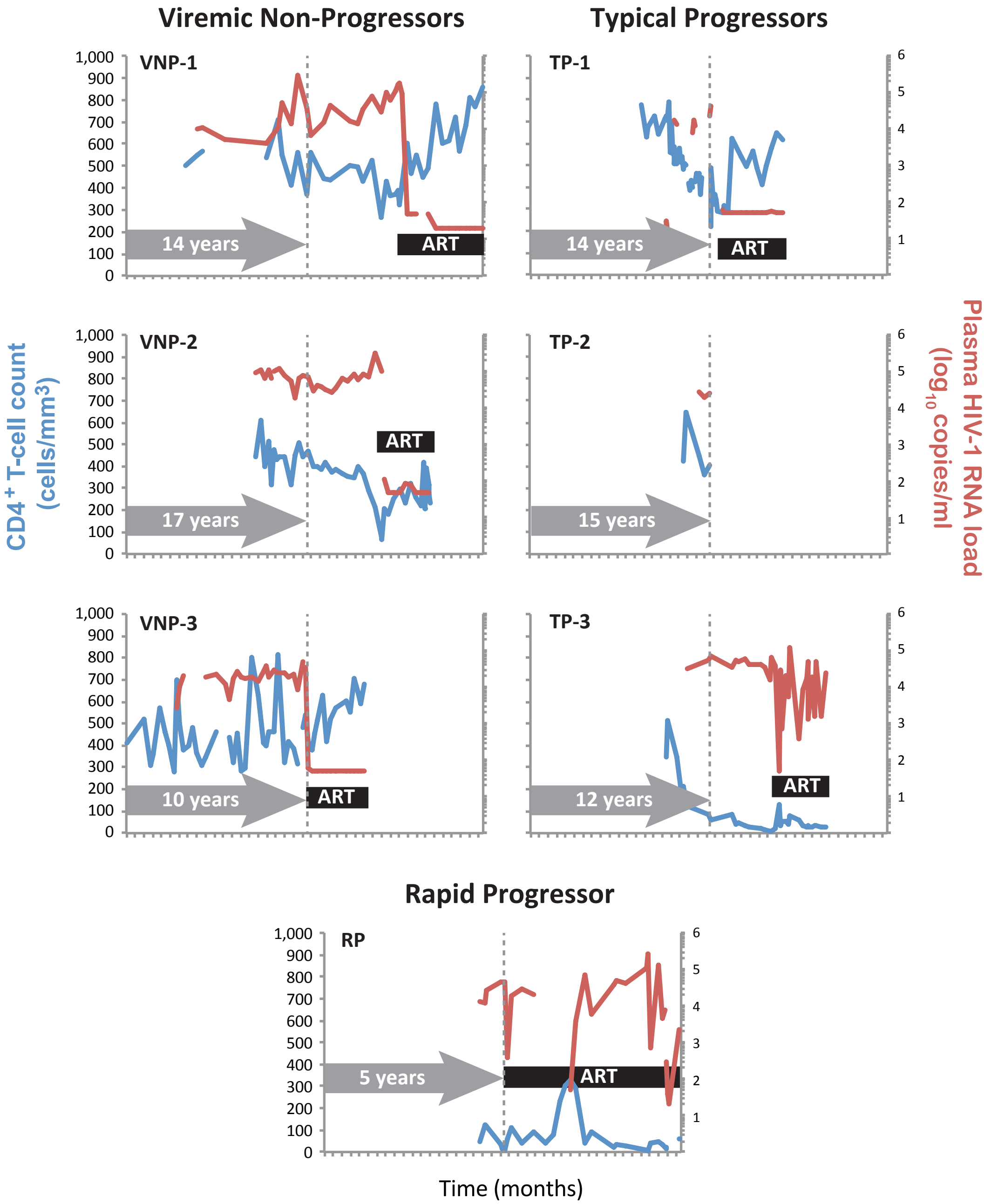

Supplementary Figure 1. CD4$^+$ T-cell counts and plasma HIV-1 RNA load values available for all seven patients described in this study. Vertical dashed lines indicate the time of sampling. Grey arrows depict the number of years since primary HIV-1 infection. The period of time the patients were under antiretroviral treatment (ART) is indicated by black boxes.
