# Supplementary material for: Impaired human immunodeficiency virus type 1 replicative fitness in atypical viremic non-progressor individuals
Source: AIDS Res Ther. 2017 Mar 20;14:15. doi: 10.1186/s12981-017-0144-0 (PMC5359922; doi:10.1186/s12981-017-0144-0)
Supplement: Supplementary file 2 — Additional file 2: Figure S2. Subtyping of the near full-length HIV-1 consensus sequences, obtained by deep sequencing, was performed using the Recombination Identification Program (RIP 3.0) from the Los Alamos HIV Sequence Database as described (1). The similarity plots show distance measurements between the query sequence (patient-derived HIV-1) and the reference HIV-1 sequences from different subtypes. [file 12981_2017_144_MOESM2_ESM.pdf]

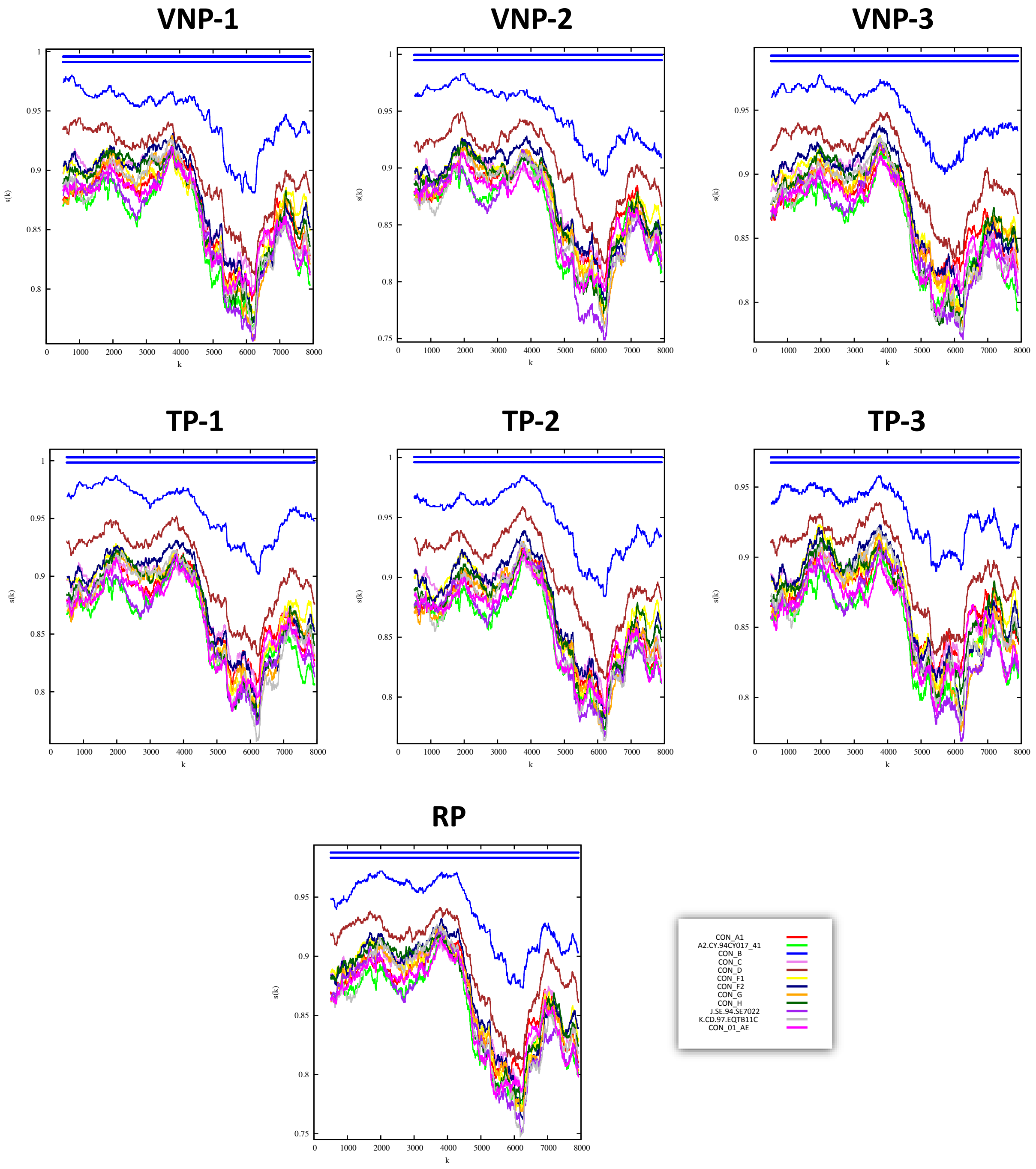

Supplementary Figure 2. Subtyping of the near full-length HIV-1 consensus sequences, obtained by deep sequencing, was performed using the Recombination Identification Program (RIP 3.0) from the Los Alamos HIV Sequence Database as described (1). The similarity plots show distance measurements between the query sequence (patient-derived HIV-1) and the reference HIV-1 sequences from different subtypes.

1. Siepel A.C., Halpern A.L., Macken C, and Korber B.T. (1995). A computer program designed to screen rapidly for HIV type 1 intersubtype recombinant sequences. AIDS Res Hum Retroviruses 11:1413-6
