# Supplementary material for: Impaired human immunodeficiency virus type 1 replicative fitness in atypical viremic non-progressor individuals
Source: AIDS Res Ther. 2017 Mar 20;14:15. doi: 10.1186/s12981-017-0144-0 (PMC5359922; doi:10.1186/s12981-017-0144-0)
Supplement: Supplementary file 3 — Additional file 3: Figure S3. Hierarchical clustering analysis of single nucleotide polymorphisms (SNPs) in all HIV-1 coding regions plus the long-terminal regions (LTRs). Dendograms were calculated using the Euclidian distance and Complete cluster methods with 1000 bootstrap iterations as described in Los Alamos HIV Sequence Database (http://www.hiv.lanl.gov.content/sequence/HEATMAP/heatmap.html). Bootstrap values >60% are indicated by an asterisk. Green and grey blocks indicate the presence and absence of SNPs, respectively. [file 12981_2017_144_MOESM3_ESM.pdf]

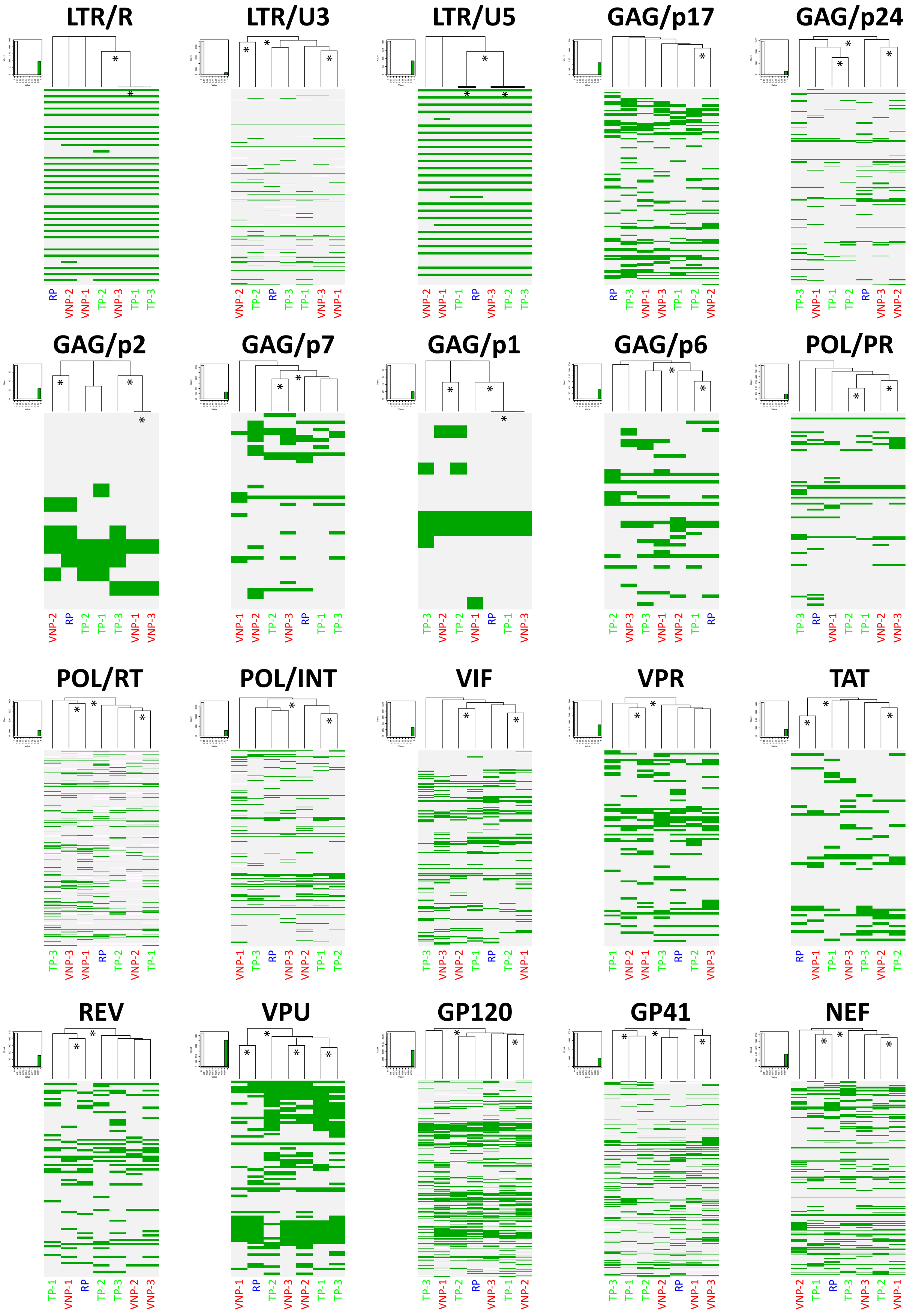

Supplementary Figure 3. Hierarchical clustering analysis of single nucleotide polymorphisms (SNPs) in all HIV-1 coding regions plus the long-terminal regions (LTRs). Dendograms were calculated using the Euclidian distance and Complete cluster methods with 1000 bootstrap iterations as described in Los Alamos HIV Sequence Database (http://www.hiv.lanl.gov.content/sequence/HEATMAP/heatmap.html). Bootstrap values >60% are indicated by an asterisk. Green and grey blocks indicate the presence and absence of SNPs, respectively.
